# Use of Toll-Like Receptor 3 Agonists Against Respiratory Viral Infections

**DOI:** 10.2174/187152111800194434

**Published:** 2011-10

**Authors:** ME Christopher, JP Wong

**Affiliations:** DRDC Suffield, Box 4000, Station Main, Medicine Hat, AB, T1A 8K6, Canada

**Keywords:** Coronavirus, influenza, innate immune system, respiratory syncytial virus, rhinovirus, poly (ICLC), Toll-lik ereceptor 3.

## Abstract

Respiratory RNA viruses are constantly evolving, thus requiring development of additional prophylactic and therapeutic strategies. Harnessing the innate immune system to non-specifically respond to viral infection has the advantage of being able to circumvent viral mutations that render the virus resistant to a particular therapeutic agent. Viruses are recognized by various cellular receptors, including Toll-like receptor (TLR) 3 which recognizes double-stranded (ds)RNA produced during the viral replication cycle. TLR3 agonists include synthetic dsRNA such as poly (IC), poly (ICLC) and poly (AU). These agents have been evaluated and found to be effective against a number of viral agents. One major limitation has been the toxicity associated with administration of these drugs. Significant time and effort have been spent to develop alternatives/modifications that will minimize these adverse effects. This review will focus on the TLR3 agonist, poly (IC)/(ICLC) with respect to its use in treatment/prevention of respiratory viral infections.

## INTRODUCTION

Response to pathogen infection is multifaceted, employing both the innate and the adaptive (acquired) immune systems. Exploitation of the adaptive immune system as a defence against viral infections has been used extensively since 1796, when Edward Jenner inoculated James Phipps with cowpox and then later challenged him with variolous material [1]. Vaccination utilizes the adaptive immune system to mount a specific immune response in the aim of disease prevention, and its use has resulted in eradication of certain diseases from the population, most notably small pox. Production of effective vaccines can be limited by genetic instability leading to reversion of an attenuated strain to a virulent strain with resultant infection in the vaccinated individual, heat sensitivity of the vaccine necessitating a requirement for refrigeration which is not always possible in developing countries, the lack of good antigenic proteins for subunit vaccines, among other limitations [2, 3]. 

Utilization of the innate immune system to non-specifically respond to viral infections has been investigated more recently. Mucosal surfaces serve as the entry sites for the majority of infectious pathogens, including respiratory viruses, and provide the first line of defence against infection [4]. During the early stages of infection, the immune response is non-antigen specific, involving natural killer (NK) and natural killer T (NKT) cells, which, through the activation of antigen presenting cells (APCs), indirectly respond to danger signals derived from invading pathogens. Certain conserved molecular structures present in pathogens (pathogen-associated molecular patterns, PAMPs), such as bacterial lipoprotein and lipopolysaccharide, are recognized by innate immune cells via pathogen recognition receptors (PRRs). Cells are activated upon recognition of PAMPs by PRRs, and trigger the production of cytokines necessary for the development of effective immunity.

The Toll-like receptors (TLRs) are a family of PRRs that are highly conserved from Drosophila to humans, with humans possessing at least 10 different TLRs. TLRs consist of an extracellular leucine-rich repeat and a cytoplasmic Toll/interleukin (IL)-1 receptor (TIR) homolog domain [5]. Although TLRs share structural and functional similarities they exhibit different patterns of expression and respond to different PAMPs. Peptidoglycans are recognized by TLR2, double-stranded (ds)RNA by TLR3 [6-8], lipopolysaccharide by TLR4, single-stranded (ss)RNA by TLR7 and 8 [9] and unmethylated deoxycytidyl-deoxyguanosinyl (CpG) dinucleotides by TLR9 [5-7, 10]. Upon detection of viral infection through PRRs, dendritic cells (DCs) become activated and traffic to lymph nodes, where they present viral antigens, induce production of interferons (IFNs) (IFN-α, -β, -γ and -λ) and cytokines such as IL-12, -15 and -18 [8, 11-17], induce CD8(+) cytotoxic T lymphocytes (CTL), activate NK cells and induce proliferation of CD4(+) T cells including Th1, Th2, Treg and Th17 cells [8, 17-22]. Activated CD4(+) T cells activate virus-specific B cells, resulting in antibody production and an adaptive immune response. 

## TLR3 AND RESPIRATORY VIRUSES

Double-stranded RNA is produced during the replication cycle of most viruses and triggers antiviral immune responses through TLR3 or retinoic acid-inducible gene I (RIG-I)/melanoma differentiation-associated gene 5 (MDA5), the latter are helicases that bind the cytoplasmic dsRNA generated during viral replication [23, 24], Fig. (**1**). In mammals, dsRNA interaction with RIG-I/MDA5 triggers IFN regulatory factor-3 (IRF-3) activation followed by type I IFN induction through the mitochondrial antiviral signaling (MAVS)/ IFN-β promoter stimulator 1 (IPS-1) adapter [24, 25]. RIG-I is essential for triggering innate immune defenses against respiratory syncytial virus (RSV) and influenza viruses [26]. 

TLR3 is localized in the endoplasmic reticulum of unstimulated cells, moves to dsRNA-containing endosomes in response to dsRNA, and colocalizes with c-Src tyrosine kinase on endosomes containing dsRNA in the lumen [27]. TLR3 is constitutively expressed in nasal epithelial cells [28], alveolar and bronchial epithelial cells [29], T, NK [8, 21] and mast cells [17], and its expression is positively regulated by influenza A virus, rhinovirus, RSV and by dsRNA [29-33]. Demonstration that the more differentiated airway epithelial cells have increased resistance to rhinovirus infection suggests an important role for TLR3 in some respiratory tract infections [28]. 

All TLRs, except TLR3, share a common signalling pathway that depends on myeloid differentiation primary response gene 88 (MyD88) [24, 34], Fig. (**1**). Stimulation of TLR3, however, results in the recruitment of TIR-containing adaptor molecule 1 (TICAM)/TIR-domain-containing adapter inducing IFN-β (TRIF). The dissociation of TRIF activates a complex consisting of the kinases IκB kinase inducible (IKKi or IKKε), TANK-binding kinase-1 (TBK1) and the scaffolding protein tumour necrosis factor (TNF) receptor-associated factor (TRAF)-3. This leads to the activation of IRF-3 and IRF-7 and the expression of IFN-inducible genes such as those encoding IFN-α, IFN-β, and genes involved in T cell stimulation such as CD40, CD80 and CD86. TRIF recruitment is also responsible for activation of the nuclear factor κB (NFκB) through interaction with the TRAF-6 complex [24, 34]. This leads to the transcription of inflammatory mediators such as TNF-α and the stabilization of inflammatory response protein mRNAs through the AU-rich elements in the 3’ untranslated region [34]. MyD88, although not part of the signalling pathway for TLR3, may play a role in the negative regulation of TLR3-induced mucin production [35].

TLR3 is unique among the TLRs in that it is able to trigger apoptosis through a pathway distinct from the mitochondrial apoptotic pathway. TLR3 mediated apoptosis occurs through its interaction with TRIF, which can then interact with Fas-associated death domain protein (FADD) and caspase-8, Fig. (**1**). This induction of apoptosis by TLR3 is an important contributor to host defence since it limits the spread of viral infection [24].

Viruses that infect the respiratory system cause effects ranging from nuisance (i.e. common cold) to severe (i.e. severe acute respiratory syndrome (SARS), H5N1 influenza), with many viral infections being exacerbated by underlying medical conditions. The majority of respiratory viruses are RNA viruses that can be recognized by TLR7 (recognizes ssRNA), TLR3 (recognizes dsRNA in endosomes) and RIG-I/MDA5 (recognizes dsRNA in cytosol). RNA viruses have either a (+)sense (Group 4) or (-)sense (Group 5) ssRNA genome. Application of TLR3 agonists to treatment/ prophylaxis of two (+) and two (-)sense RNA viruses, each belonging to a different family, will be discussed. 

### Influenza Virus

Influenza virus is a segmented (-)sense RNA virus of the *Orthomyxoviridae* family. Influenza viruses are transmitted primarily through aerosol inhalation of contagious droplets generated by sneezing or coughing, or through direct contact with an infected person. Influenza pandemics occur when a novel strain of influenza virus causes a severe epidemic that spreads over several geographical locations and affects an exceptionally high proportion of the population in those locations. Past human influenza virus pandemics have been caused by influenza A H1N1 (1918-1920 and 2009), H2N2 (1957-1960) and H3N2 (1968-1972) subtypes [36]. In recent years, highly pathogenic avian influenza A viruses with subtypes H5N1, H6N1 [37], H7N2 [38], H7N3 [38, 39], H7N7 [39-42] and H9N2 [39, 43] have caused outbreaks in poultry in Africa, Europe, the Middle East, Asia and North America and all have pandemic potential [44, 45]. Fortunately the 2009 pandemic influenza A H1N1 strain caused relatively mild disease in the majority of people infected.

Influenza virus infection induces expression of IL-1β, -2, -6, -8, -18, TNF-α, Fas ligand, IRF-1 and IFN-α and -β [29, 46, 47]. Regulated upon activation, normal T-cell expressed and presumably excreted (RANTES), dsRNA dependent protein kinase R (PKR), indolamine 2,3-deoxygenase, intra-cellular adhesion molecule (ICAM)-1, 2’-5’-oligoadenylate synthetase and TLR3 are also induced [29, 46, 47]. IL-18 is involved in controlling influenza virus replication in the lung, especially at early stages of infection, through activation of innate immune mechanisms including IFN production and NK cell activation [48]. IL-2, produced by T cells specific for influenza A virus, is involved in T cell dependent IFN-γ production by NK cells, suggesting that at an early stage of recurrent viral infection, NK-mediated innate immunity to the virus is enhanced by pre-existing virus specific T cells [49]. 

Influenza viral infection induces necrosis in infected epithelial cells and apoptosis in monocytes/macrophages, the latter being mediated by either perforin [50] or IFN-β and regulated by PKR and IRF-1 [46]. Human DCs that capture dead cells containing influenza virus produce cytokines, undergo maturation and expand CD4(+) T helper cells but fail to elicit CD8(+) cytotoxic T cell responses against antigens from the dead cells [51]. A recent study demonstrated that chickens infected with H9N2 strains were resistant to H5N1 influenza virus challenge. Protective immunity was closely related to the percentage of CD8(+) T cells expressing IFN-γ in the lung, rather than in the spleen, suggesting that pulmonary cellular immunity may be very important in protecting naïve natural hosts against lethal influenza viruses [52].

During a single-cycle infection, human influenza viruses preferentially infect nonciliated epithelial cells, whereas avian viruses, as well as the egg-adapted human virus variant with avian virus-like receptor specificity, primarily infect ciliated cells. This correlates with the predominant localization of receptors for human (α2,6-linked sialic acids) and avian (α2,6-linked sialic acids) viruses on nonciliated and ciliated cells, respectively [53]. Sialic acid linked to galactose via α2,3 glycosidic bonds, is a cellular receptor located in the eye, which may account for the ocular tropism exhibited by some zoonotic avian influenza A viruses [38].

Some strains of influenza virus result in hyperinduction of pro-inflammatory and apoptotic cytokines. This resulting “cytokine storm” may lead to lung injury and multiple organ failure. Since the “cytokine storm” is mediated by a functional immune system, those with normal immune system functions are more susceptible to severe and adverse effects of infections by these strains, which include avian influenza H5N1. There are significant differences in cytokine responses between H5N1 (A/HK/483/97, A/Vietnam/1194/04 and A/Vietnam/3046/04) and H1N1 influenza viruses, primarily in relation to TNF-α, IFN-β, IL-6, IP-10 and RANTES which are elevated post-infection (p.i.) in human type II pneumocytes, but significantly more so in H5N1 infected cells, with more recent H5N1 viruses from Vietnam (H5N1/04) being more potent at inducing IP-10 [54-56]. Inactivation of the virus by UV irradiation prior to infection of alveolar epithelial cells abolished cytokine induction suggesting that virus replication was required for cytokine induction [55]. 

Influenza H5N1 infection in birds is systemic, characterized by hemorrhage and pulmonary edema resulting from virus replication and stimulation of vascular endothelial growth factor in the alveolar endothelium [57, 58]. Systemic infection has also been observed in cynomolgus macaques and humans [59, 60]. In mice infected with influenza H5N1/ 97, virus-infected cells initially appeared in the respiratory tract and later could be detected in neurons, glial and ependymal cells of the central nervous system [61]. 

H5N1 influenza viruses can be further classified into high and low pathogenicity viruses. The difference in host response to the lethal and nonlethal H5N1 influenza virus is likely due to the viral nonstructural (NS) protein [54, 56, 62, 63] which binds dsRNA, effectively shielding it from the immune system [64], and has been shown to inhibit apoptosis [65], RIG-I, IPS-1 and nuclear translocation of IRF3 [64], Fig. (**1**). Mice infected with a lethal (A/Hong Kong/483/97) H5N1 influenza virus had a significant decrease in the total number of circulating leukocytes (primarily lymphocytes) as early as 2 days p.i. and a reduction in the number of CD4(+) and CD8(+) T cells [66]. IL-1β, IFN-γ and macrophage inflammatory protein (MIP)-1α in lung and lymphoid tissue were elevated in mice infected with either a lethal (A/Hong Kong/483/97) or nonlethal (A/Hong Kong/486/97) H5N1 influenza virus, although the degree of elevation was lower in mice infected with the lethal strain [67-69]; whereas, TNF-α and MIP-2 levels were elevated in a similar manner by both strains [68, 69]. It has been suggested that TNF-α may contribute to early disease severity whereas IL-1 may play a role in viral clearance late in H5N1 infection [67]. Mice infected with the lethal H5N1 influenza virus HK483 also had increased concentrations of IL-1β, TNF-α, IFN-γ, MIP-1α and MIP-2 in the brain, and apoptosis in the spleen and lung [66]. Although A/HK/483/97 infected mice showed no evidence of virus-induced encephalitis, the local synthesis of TNF-α or IL-1 within the brain could contribute to anorexia, weight loss and death [70]. The lethal H5N1 influenza virus appears to possess the capacity to limit the induction of immune responses by targeting and destroying lymphocytes, resulting in aberrant production of cytokines in serum and tissues. In addition to the cytokine storm, systemic viral dissemination and alveolar flooding due to inhibition of cellular sodium channels contribute to the lethality of influenza H5N1 disease [55, 56, 60, 71-73].

### Respiratory Syncytial Virus

RSV is a (-)sense, enveloped RNA virus of the *Paramyxoviridae* family. RSV is a ubiquitous pathogen causing upper respiratory infections in healthy adults, bronchiolitis and pneumonia in young children, exacerbations of disease in patients with chronic obstructive pulmonary disease and asthma, and life-threatening pneumonia in immunosuppressed patients. RSV infection increases the responsiveness of airway epithelial cells to subsequent bacterial ligands [31]. RIG-I is responsible for the early recognition of RSV dsRNA (within 12 hours of infection) and TLR3 is important for later responses. TLR3 induction by RSV is mediated by RIG-I-dependent IFN-β secreted from infected airway epithelial cells and is mediated by IFN-stimulated response element (ISRE) and signal transducer and activator of transcription (STAT) sites in its proximal promoter [74]. Addition of an inhibitor of PKR demonstrated no effect on IL-8 induction following RSV infection but a significant difference following polyriboinosinic-polyribocytidylic acid (poly (IC)) treatment, suggesting that PKR does not play a role in primary RSV-induced inflammation but does play a role in poly (IC)-induced secondary inflammation [31]. RSV NS1 and NS2 proteins block IFN signaling by stimulating proteasomal degradation of STAT2 and interfering with the TLR3 and RIG-I pathways, Fig. (**1**) [75]. RSV also inhibits viral-induced apoptosis [76]. Use of knock-out (KO) mice has shown that TLR2 and TLR6 signaling in leukocytes can activate innate immunity against RSV (by inducing TNF-α, IL-6, chemokine (C-C) ligand (CCL) 2/monocyte chemo-attractant protein (MCP)-1, and CCL5/RANTES) and control viral replication. TLR2/RSV interactions also promote neutrophil migration and DC activation within the lung [77].

Microarray analysis of the pulmonary innate immune response to RSV demonstrated that induction of most IFN-, cytokine-, chemokine- and PRR-related genes was MAVS dependent and MyD88 independent. One day after RSV infection, only wild type (WT) mice exhibited pulmonary dysfunction, possibly attributable to inflammatory cytokine responses that cause airway obstruction. Serum anti-RSV antibody responses were significantly attenuated in mice lacking either MAVS or MyD88 and were reduced even further in double KO mice suggesting that both MAVS and MyD8 play an important role in the generation of anti-RSV antibodies, in contrast to antibody production in response to influenza which is MyD88 dependent and MAVS independent [78].

### Coronavirus

Coronavirus (Co-V) is a non-segmented (+)sense RNA virus of the *Coronaviridae* family. Coronaviruses are transmitted by aerosols of respiratory secretions, the fecaloral route and mechanical transmission. Most viral growth occurs in epithelial cells, however, organs such as the liver, kidneys, heart, eyes or other cell types (i.e. macrophages) may occasionally be infected. Most infections are localized to the epithelium of the upper respiratory tract causing a mild, self-limited disease (classical ‘cold’ or upset stomach), occasionally causing enteric infections (primarily in infants <12 months), and rarely causing neurological syndromes. Coronavirus infection is very common in children, less common in adults, occurs worldwide and exhibits a strongly seasonal incidence of infection. Re-infections recur through-out life, implying multiple serotypes (at least four are known) and/or antigenic variation, hence the prospects for immunization appear bleak. 

SARS is a form of viral pneumonia where infection encompasses the lower respiratory tract. Clinical symptoms include fever, dry cough, dyspnea (shortness of breath), headache, and hypoxemia. Typical laboratory findings include lymphopenia and mildly elevated aminotransferase levels, indicative of liver damage. Death may result from progressive respiratory failure due to alveolar damage. The typical clinical course of SARS involves an improvement in symptoms during the first week of infection, followed by worsening during the second week. Studies indicate that this worsening may be related to the patient’s immune responses rather than to uncontrolled viral replication [79]. TLR signaling is important for SARS-Co-V recognition by the innate immune system since C57BL/6 mice which are resistant to SARS-Co-V strain MA15, are susceptible when MyD88 is genetically deleted [80]. SARS-Co-V is an inefficient activator of alveolar macrophages and respiratory DCs, therefore impairing anti-viral responses such as IFN-γ production, endocytosis and T cell responses [81]. SARS-Co-V protein 7a inhibits translation of various cellular RNAs and is involved in induction of cellular stress responses, including apoptosis [82]. SARS-Co-V NSP15, on the other hand, inhibits apoptosis thus facilitating viral immune evasion and virus propagation [83], Fig. (**1**). Analysis of splenocytes from WT mice determined that non-T cells were responsible for the high TNF-α and IFN-γ levels present following infection with coronavirus strain MHV-A59, which primarily infects the liver and brain. These high cytokine levels could be abrogated by addition of T cells, with cell-to-cell contact being required. Consequently, as would be expected, T-cell- or lymphocyte-deficient mice infected with this strain exhibited symptoms of cytokine storm [84].

### Rhinovirus

Rhinovirus is a non-segmented (+)sense RNA virus of the *Picornaviridae* family. Rhinoviruses have a growth optimum of 33°C, proliferating in the ciliated epithelium of the upper respiratory tract where damage to these cells results in symptoms and predisposition to secondary bacterial infections. Interestingly, more differentiated airway epithelial cells, which have higher TLR3 expression, have increased resistance to rhinovirus infection [28]. Rhinovirus replication also induces β-defensins-2 and -3 [85] and mucin production in primary human epithelial cells and cell lines, with induction being dependent on TLR3 and mucin production being negatively regulated by MyD88, and only partially dependent on TRIF [35]. 

Rhinovirus infection is the most common cause of acute exacerbations of inflammatory lung diseases, such as asthma and chronic obstructive pulmonary disease, where it provokes steroid refractory and abnormally intense neutrophilic inflammation that can be life threatening [28, 30, 35, 86, 87]. Rhinovirus strain 16 (RV16) infection rapidly promotes TLR3-mediated induction of epidermal growth factor receptor (EGFR) ligands and utilizes EGFR signaling to increase IL-8 and ICAM-1 levels. This coupling of antiviral defense machinery (TLR3) and the major epithelial proliferation/ repair pathway may play an important role in viral-induced airway remodeling and airway disease exacerbation [35] and suggests that targeting EGFR may provide a selective therapy that dampens neutrophil-driven inflammation without compromising essential antiviral pathways mediated by PRRs [86].

Rhinovirus replication increases expression of TLR3 mRNA and TLR3 protein on the cell surface and blocking TLR3 leads to a decrease in IL-6, CXCL8, and CCL5 in response to poly (IC) but an increase following rhinovirus infection. This demonstrates an important functional requirement for TLR3 in the host response against rhinovirus infection, indicates that poly (IC) is not always a good model for studying the biology of live viral infections [30] and suggests that poly (IC) could be utilized therapeutically because of differences in mechanisms of action. The RIG-I/MDA-5 pathway is important in the induction of type I IFN following rhinovirus infection, however, rhinovirus is able to inhibit IPS-1 in order to evade this arm of the immune response [88], Fig. (**1**).

## DRUGS TARGETING TLR3

Synthetic dsRNAs, at least 40-50 base pairs in length [89], including poly (IC) alone or stabilized with poly-L-lysine and carboxymethyl cellulose (poly (ICLC)) and poly-adenosinic-polyuridylic acid (poly (AU)) have been used as molecular mimics for viral dsRNA [14, 19, 90]. A comparative study in mice using various TLR3 mimics determined that IFN induction was highest with poly (ICLC) and lowest with poly (AU) but splenocyte cytotoxicity was significantly higher with poly (ICLC) than with poly (AU) [91]. 

Poly (ICLC) has been shown to up-regulate IL-1, -10, -12b, -15, IFN-β, -γ and TNF-α and to down-regulate IL-2, -4 and -5 in lung tissue of treated mice [33]. The signaling pathway invoked by poly (IC) administration varies with the cell type responding. Intraperitoneal (i.p.) administration of poly (IC) to WT, TRIF KO, IPS-1 KO and double KO demonstrated that serum IL-6 induction was dependent on both IPS-1 (primarily) and TRIF pathways, whereas IL-12p40 induction was entirely dependent on the TRIF pathway. In the spleen, poly (IC) induction of IFN-α, -β and –γ and IL-6 was dependent on IPS-1, whereas IP-10 and RANTES were dependent on both IPS-1 and TRIF. In DC, IFN-β and IL-12p40 production was dependent on both IPS-1 and TRIF. Additionally, IPS-1 KO mice had severe defects in poly (IC)-enhanced antibody production whereas TRIF KO mice displayed modestly reduced antibody production [92]. In intestinal epithelial cells, poly (IC) activated IRF-3 dimerization and phosphorylation, increased activity of ISRE, induced IFN-β and up-regulated the expression of IFN-regulated genes in a RIG-I and IPS-1-dependent manner with TLR3 signaling not being involved, even though it was expressed in these cells [93]. However, gender differences have been noted in human and Rhesus monkeys, with males having consistently and significantly higher IFN responses than females [94, 95]. Thus, the antiviral activity of IFNs and/or activation of NK cells induced by dsRNA may result in non-specific antiviral defences against a number of viral agents, and may therefore provide a broad-spectrum antiviral effect against viruses, regardless of strain, subtype and drug resistance. 

Poly (IC) and/or poly (ICLC) has been shown to be effective in rodents, primates and the marine crustacean *Litopenaeus vannamei* [96]. Protection has been observed against influenza virus [90], Rift Valley fever virus [16], rabies virus [97], Punta Toro virus [98], Herpes simplex virus type 2 [99], western equine encephalitis virus [100], Venezuelan equine encephalomyelitis virus [101], yellow fever virus [102], SARS-Co-V [81], RSV [103], white spot syndrome virus and Taura syndrome virus [96]. Poly (IC) has also been shown, through its effects on NK cells, to eliminate the histological lesions of graft-versus-host disease in mice [104].

Influenza NS1 protein sequesters dsRNA, binds to RIG-I and inhibits downstream activation of IRF-3, preventing the transcriptional induction of IFN-β, Fig. (**1**); thus poly (IC), by way of TLR3 signaling, may act to overcome this NS1 inhibition [64]. In mice, prophylactic treatment with two intranasal (i.n.) doses of 1 mg/kg/dose poly (ICLC) provided complete protection against lethal influenza A/PR/8/34 (H1N1) or A/Aichi/2/68 (H3N2) viral challenge [90, 105] and partial protection (63-75%) against A/H5N1/chicken/ Henan (clade 2) [106]. This suggests that poly (IC) did not add to the cytokine storm associated with the mortality of influenza A H5N1 viruses. Mice given one poly (ICLC) dose 20, 16, 14 or 12 days prior to influenza viral challenge and a second dose one day prior to challenge had survival rates of 0, 40, 80 and 100%, respectively [90]. Mice that received liposome-encapsulated poly (ICLC) 21 and 1 day prior to virus challenge were fully protected, thus increasing the short-term window of prophylaxis provided by poly (ICLC) [105]. Poly (ICLC) may thus provide short-term prophylaxis against influenza as suggested by the multi-day window of protection [90] and by the observation that NK cell activity remains elevated for nine and six days post-poly (ICLC) treatment in liver and blood/spleen, respectively [65]. Poly (AU) also increased NK cell activity in the liver but doses approximately 10-fold higher than those used with poly (ICLC) were required [20]. Treatment with a single 20 µg dose of poly (IC) 18-24 hours prior to infection with SARS-Co-V protected mice from lethal disease, with minimal weight loss, enhanced CD86 and CD40 up-regulation of alveolar macrophages, generation of early and robust antigen-specific T cell responses and increased type I IFN production, even though SARS-Co-V is only moderately sensitive to the effects of IFN [81]. Similarly, when poly (ICLC) was given 24 hours prior to RSV infection, marked reduction in viral titres and amelioration of clinical illness and reduced inflammation were observed. However, when poly (ICLC) was given 48 hours post-RSV exposure, poly (ICLC) mediated IFN-α production was completely suppressed [103].

Poly (ICLC) administered post-influenza virus exposure was also less effective than when administered prophylactically. Mice treated with two intravenous (i.v.) doses (1 mg/kg/dose) of poly (ICLC) 8 and 48 hours p.i. showed a small increase in survival (40%) compared to untreated control mice and a single post-exposure treatment was found to be almost completely ineffective [90]. Post-exposure efficacy has been shown in mice infected with Punta Toro virus [98] and West Nile virus when treatment started one day prior to infection and continued every 48 hours until 5 days p.i. [107]. When treatments were delayed to 4-6 hours before viral challenge, efficacy was greatly reduced [107].

Poly (IC) has also been used as an adjuvant with split-product influenza vaccines. Treatment with vaccine plus poly (IC) rapidly up-regulated TLR3 expression in the nasal-associated lymphoid tissue, up-regulated IL-4 and IL-12p40, and induced IFN-α, -β and -γ [108]. When mice were subsequently challenged with influenza A/PR/8/34 (H1N1), cross-protection, partial protection or no protection was observed in those immunized with various H1N1 virus vaccines (A/PR/8, A/Beijing, A/Yamagata), heterologous influenza A vaccines (A/Guizhou – H3N2) or influenza B vaccines (B/Ibaraki, B/Yamagata, B/Aichi), respectively [109]. T-cell activation and increased IFN-γ production was observed only in mice immunized with homologous antigens [108]. Poly (ICLC) has been shown to be an effective adjuvant when co-administered with retinoic acid, a vitamin A metabolite [110], chloroquine (for *Plasmodium yoelii nigeriensis* infections) [111], anti-Semliki Forest virus hyperimmune serum [112] and IFN-α/β (inhibited flavivirus replication) [113]. For clinical use, poly (IC) may be one of the most appropriate agents to generate stable mature DCs. These mature DCs might generate *in vivo* effective immune responses after injection, because they retain the ability to secrete bioactive IL-12 after CD40 ligation [114].

## ADVERSE EFFECTS OF TLR3 AGONISTS

The potential of poly (ICLC) as an anti-viral agent is, however, limited by its intrinsic toxicity. Toxicity of poly (ICLC) is affected by the route of administration with subcutaneous (s.c.) administration being tolerated well in rabbits [115] and mice (unpublished observations) and intratracheal administration being well tolerated in mice [116]. Intramuscular (i.m.), i.p., i.n. or i.v. administration results in variable levels of toxicity depending on the animal species and doses being used [54]. Mice given three i.n. doses of 100 µg poly(IC) 24 hours apart demonstrated signs of lung inflammation (increased neutrophils in bronchio-alveolar lavage fluid, increased cytokine levels, cellular infiltration in the lungs and decreased lung function), which was milder than the reaction seen in TLR3 KO mice [117]. In clinical trials, patients receiving multiple therapeutic doses of poly (ICLC), i.v. or i.m., exhibited serious toxic reactions including hypotension, fever, anemia, leukopenia, thrombocytopenia, nausea, injection site inflammation and, in multiple sclerosis patients, neurological dysfunction [15, 109, 118-128].

Contradictory results regarding poly (IC) and auto-immune disorders have been reported. In auto-immuneprone mice (MRL), poly (IC) administration induced chronic pancreatitis [129] and aggravated lupus nephritis [130]. TLR3 up-regulation has also been reported to promote proinflammatory autoimmune responses, islet destruction, diabetes and hepatic disease [131-133]. In contrast, systemic application of poly (IC) or R-848 (a TLR7 ligand) during the sensitization phase abolished all features of experimental asthma, including airway hyperresponsiveness and allergic airway inflammation. Additionally, administration to animals with already established primary allergic responses revealed a markedly reduced secondary response following allergen aerosol rechallenge [134]. Poly (IC) administration suppressed immune complex-mediated experimental arthritis in mice with suppression dependent on type I IFNs that inhibited synovial cell proliferation and inflammatory cytokine production [135]. However, other studies of rheumatoid arthritis patients suggested that poly (IC) upregulated TLR3 expression and activation in synovial fluid fibroblasts and stimulated proinflammatory gene expression, suggesting that RNA released from necrotic cells might act as an endogenous TLR3 ligand in rheumatoid arthritis [136]. Intra-articular poly (IC) administration induced arthritis, mediated by IL-1 receptor (IL-1R) signalling, as early as 3 days post-administration [137]. Thus the potential link between TLR up-regulation and autoimmunity emphasizes the need for caution in using TLR agonists as new therapies or vaccine adjuvants [131].

Attempts to improve safety and efficacy of poly (ICLC) have focused on optimization of dosage and treatment regimes, encapsulation within liposomes, modification of poly (ICLC), and co-administration of agents that mitigate cytokine-mediated adverse reactions [54]. In patients with advanced cancer, optimal poly (ICLC) administration regimes were found to be those administered on an alternate-day schedule with gradual dose escalation. In these studies the maximum tolerated dose varied over a several hundredfold dose range [119]. 

In mice, poly (ICLC) administration resulted in loss of up to 10% of the total body weight and hypothermia of up to 2°C [101]. Encapsulation of poly (ICLC) within cationic liposomes composed of phosphatidylcholine, cholesterol and stearylamine completely mitigated the toxicity (as determined by absence of weight loss and changes in body temperature) observed with free poly (ICLC) when administered i.v. [54, 105]. Liposome-encapsulation of poly (ICLC) did not reduce weight loss following i.n. administration, but it did reduce the magnitude and duration of body temperature reduction following influenza infection [105]. Liposomes have been shown to accumulate at sites of infection [15], possibly concentrating the encapsulated drug at the diseased site and thereby minimizing the exposures of healthy organs and tissues to the drug, resulting in a gradual and sustained release of poly (ICLC), thereby avoiding rapid systemic elevation of drug levels observed with some routes of administration. It is unclear whether the significant reductions in the toxicity of poly (ICLC) provided by liposomes will result in corresponding decreases in clinical side effects seen in human patients. 

Modifying poly (IC) has resulted in variable success. Modifications have included the use of lower molecular weight molecules and thiolation. In rabbits, a low molecular weight poly (ICLC) induced high IFN responses and ameliorated hypotensive responses, however, it also induced high fevers [115]. Optimal antiviral and antiproliferative activities were observed when thiolation of the five position of the cytosine base on the poly (C) strand was at 7.4% [138]. These results suggest that further study into modification of poly (ICLC) may be advantageous for the elimination / reduction of toxic side effects.

Mitigation of cytokine-mediated adverse reactions by hydrocortisone treatment prior to or following i.v. poly (ICLC) administration reduced both the hypotensive responses and IFN induction in rabbits [115]. The IL-1R signalling pathway, stimulated by poly (ICLC) is implicated in increased plasma IL-6 concentrations. Male rats given IL-1 receptor agonist (IL-1ra) prior to poly (IC) administration had elevated plasma TNF-α, but not IL-6, concentrations and a reduction of fever [139]. 

## IMPLICATIONS FOR USE OF TLR3 AGONISTS AGAINST CYTOKINE STORM

In humans, the highly pathogenic influenza A H5N1 (HPIA) virus mediates a cytokine storm characterized by hypercytokinemia with uncontrolled production of IFN-β, IP-10, RANTES, IL-6, TNF-α and numerous chemokines in the lung [54]. In mice, decreased CD4(+) and CD8(+) T cells, apoptosis of lymphocytes in the spleen and lung and detection of cytokines in the brain are associated with influenza H5N1 pathology. Furthermore, the NS1 protein of HPIA virus inhibits IRF3 and the general IFN signaling pathway, thus mediating evasion of the hosts antiviral IFN-mediated defences [64]. Theoretically, for non-specific immune stimulators to be effective in influenza H5N1 viral infection, they should overcome the insensitivity to IFN and should prevent apoptosis of lymphocytes without contributing further to cytokine dysregulation. Prevention of apoptosis of T cells could potentially increase TNF-β, IFN-γ, IL-2, -3, -4, -5, -9, -10 and -15. Preferably, TNF-α would not be induced by the non-specific immune stimulator as it is already highly induced by influenza A H5N1 viral infection, although mice deficient for TNF-α or its receptors had no reduction in mortality when infected with A/Vietnam/ 1203/04 (H5N1) suggesting that TNF-α alone is not responsible for the increased mortality of H5N1 influenza viruses [73]. Inhibition of the cytokine response by corticosterone, the natural mouse glucocorticoid, was not sufficient to prevent death, regardless of when the drug was administered, [73] suggesting that factors other than the cytokine storm are important for lethality. 

Treatment of mice with liposome-encapsulated poly (ICLC) has been evaluated for efficacy against influenza A/H5N1/chicken/Henan. Mice given one lethal dose 50 (LD_50_) of the virus had a 50% survival rate whereas those given two doses of liposome-encapsulated poly (ICLC) had a 100% survival rate. When the virus dose was increased to 4 LD_50_, survival of treated mice decreased to 63% whereas the untreated mice succumbed to infection [54, 106]. This demonstrates that non-specific immune stimulators may be an effective prophylaxis against a pandemic strain of influenza.

Similar to influenza H5N1 virus, RSV is also a poor inducer of IFN-α/β and is partially resistant to IFNs antiviral activity. When poly (ICLC), an IFN-α inducer, was given before RSV infection, mice had a milder disease and / or faster recovery with increased IFN production and reduced viral replication [103]. However, when poly (ICLC) was administered 48 hours post-RSV infection, IFN-α production was almost completely inhibited [103]. 

Both the innate and adaptive immune systems are affec-ted by aging. In the innate immune system, the functions of NK cells, macrophages (fewer number and less efficient antigen presentation) and neutrophils (impaired chemotaxis, degranulation and phagocytosis) are decreased with aging [140]. Age-related changes in the adaptive immune system include diminished/altered cytokine patterns (Th2 bias), reduction in clonal expansion and function of antigen-specific T and B cells and a decline in antigen-presenting cell function [140]. Humoral immunity also exhibits changes albeit to a lesser extent, particularly the diminished ability to generate high-affinity protective antibodies against infectious agents. Splenic and activated peritoneal macrophages from aged mice express significantly lower levels of all TLRs and macrophages from aged mice secrete significantly lower levels of IL-6 and TNF-α when stimulated with known TLR ligands [140]. Poly (ICLC) was able to effectively protect aged mice against lethal murine cytomegalo-virus infection and effectively induced IFN [141]. Similarly, severe combined immunodeficient (SCID) mice were almost completely protected against murine cytomegalovirus infection by poly (ICLC), and poly (ICLC) was able to induce IFN and NK cell cytotoxicity in these mice [141]. These results suggest that, although TLR3 receptors are reduced during the aging process, poly (ICLC) still has the potential to be effective [54]. 

In light of current concerns regarding viral-induced cytokine storms, poly (ICLC) appears to have potential to stimulate the innate immune system and thus provide protection. As poly (ICLC) stimulates IL-12 it would theoretically be an effective adjuvant for generating cross-protective antibodies thus expanding the number of people that could be immunized. Use of poly (ICLC) as an adjuvant requires lower doses than when used solely for prophylaxis [54], thus alleviating some of the toxicity concerns associated with their use.

## CONCLUSION

TLR3 agonists appear to be effective when used prophyl-actically against a variety of viral agents. When evaluated against agents that trigger a cytokine storm in the host (H5N1 influenza and SARS), improved survival was observed suggesting that these agonists do not contribute to the cytokine burden induced during viral infection. TLR3 agonists are also effective adjuvants. The major concerns with the use of TLR3 agonists are the toxicity associated with their use and their low efficacy when used as a therapeutic agent instead of as a prophylactic agent. Encapsulation within liposomes has been shown to be an effective means to reduce toxicity associated with the free drug. Continued work to develop regimes for TLR3 agonist therapy is required.

## Figures and Tables

**Fig. (1):**
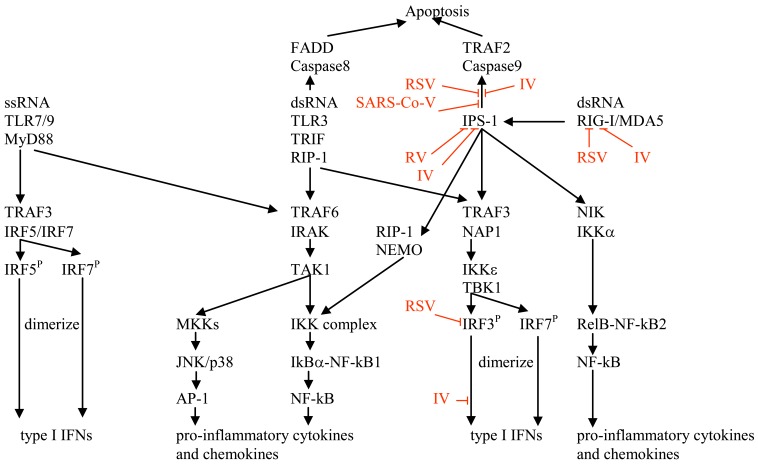
Double-stranded and single-stranded RNA signaling pathway. Double-stranded RNA is recognized in the cytosol by RIG-I or in the endosome by TLR3, whereas ssRNA is recognized by TLR7 in the endosome. Binding of dsRNA to RIG-I recruits MDA5 and the complex interacts with IPS-1 present in the mitochondrial membrane. Interaction of IPS-1 with TRAF3, NAK-associated protein 1 (NAP1), IKKε and TBK1 results in activation of transcription factors (TFs) IRF3 and IRF7 and induction of type I IFN genes. Interaction of IPS-1 with NF-κB essential modulator (NEMO), receptor-interacting protein 1 (RIP-1) and the IKK complex, or NIK and IKKα results in the release of active NF-κB and induction of various genes encoding pro-inflammatory cytokines and chemokines. In the endosome, binding of dsRNA with TLR3 results in the recruitment of TRIF and RIP-1 which can interact with TRAF6 or TRAF3. Interaction with TRAF6 and IL-1 receptorassociated kinase (IRAK) leads to the induction of pro-inflammatory cytokines through activation of TFs NF-κB and activating protein 1 (AP-1). In the endosome, binding of ssRNA to TLR7 recruits MyD88 which can interact with TRAF6 or TRAF3. The TLR7, MyD88, TRAF6 pathway intersects with the TLR3 TRAF6 pathway to induce pro-inflammatory cytokines and chemokines. The TLR7, MyD88, TRAF3 pathway results in activation of TFs IRF5 and IRF7, resulting in induction of type I IFNs [24, 34]. Apoptosis is also induced following binding of dsRNA to either TLR3 or RIG-I via FADD/Caspase 8 or TRAF2/Caspase9, respectively [24]. Influenza virus (IV) NS1 protein can inhibit the signaling pathway by binding to dsRNA preventing its recognition by RIG-I/ TLR3, inhibiting RIG-I, IPS-1, nuclear translocation of IRF3 or IPS-1-induced apoptosis [64, 65]. RSV NS2 protein can also inhibit RIG-I, IRF3 and IPS-1-induced apoptosis [75, 76]. SARS-Co-V [83] also inhibits IPS-1-induced apoptosis and rhinovirus (RV) [88] has been shown to inhibit IPS-1.

## ABBREVIATIONS

AP-1: = Activating protein 1
APC: = Antigen presenting cell
Co-V: = Coronavirus
CCL: = Chemokine (C-C) ligand
CTL: = Cytotoxic T lymphocytes
DC: = Dendritic cell
ds: = Double-stranded
EGFR: = Epidermal growth factor receptor
FADD: = Fas-associated death domain protein
HPIA: = Highly pathogenic influenza A H5N1
ICAM -1: = Intracellular adhesion molecule 1
IFN: = Interferon
IKK: = IκB kinase
IKK complex: = Complex composed of IKKα, IKKβ and NEMO/IKKγ
IKKi: = IKK inducible (also called IKKε)
IL: = Interleukin
IL-1R: = IL-1 receptor
IL-1ra: = IL-1 receptor agonist
i.m.: = Intramuscular
i.n.: = Intranasal
i.p.: = Intraperitoneal
IPS-1: = IFN-β promoter stimulator 1 (also called MAVS, CARDIF, VISA)
IRAK: = IL-1 receptor-associated kinase
IRF: = IFN regulatory factor
ISRE: = IFN-stimulated response element
i.v.: = Intravenous
IV: = Influenza virus
JNK: = *c*-Jun N-terminal kinase
KO: = Knock-out
LD_50_: = Lethal dose 50
MAVS: = Mitochondrial antiviral signaling (also called IPS-1, CARDIF, VISA)
MCP: = Monocyte chemoattractant protein
MDA5: = Melanoma differentiation-associated gene 5
MIP: = Macrophage inflammatory protein
MKK: = Mitogen activated protein kinase kinase
MyD88: = Myeloid differentiation primary response gene 88
NAP1: = NAK-associated protein 1
NEMO: = NF-κB essential modulator
NF-κB: = Nuclear factor κB
NIK: = IKKα-NF-κB-inducing kinase
NK: = Natural killer
NKT: = Natural killer T cells
NS/NSP: = Nonstructural protein
PAMP: = Pathogen-associated molecular pattern
p.i.: = Post-infection
PKR: = dsRNA dependent protein kinase R
poly (AU): = Polyadenosinic-polyuridylic acid
poly (IC): = Polyriboinosinic-polyribocytidylic acid
poly: = Poly (IC) stabilized with poly-L-lysine and (ICLC) carboxymethyl cellulose
PRR: = Pathogen recognition receptor
RANTES: = Regulated upon activation, normal T-cell expressed and presumably excreted
RIG-I: = Retinoic acid-inducible gene I
RIP-1: = Receptor-interacting protein 1
RSV: = Respiratory syncytial virus
RV: = Rhinovirus
SARS: = Severe acute respiratory syndrome
s.c.: = Subcutaneous
SCID: = Severe combined immunodeficiency
ss: = Single-stranded
STAT: = Signal transducer and activator of transcription
TAK1: = Transforming growth factor β-activating kinase
TBK1: = TANK-binding kinase 1 (also called NAK, T2K)
TF: = Transcription factor
TICAM: = TIR-containing adaptor molecule 1 (also called TRIF)
TIR: = Toll/interleukin-1 receptor
TLR: = Toll-like receptor
TNF: = Tumour necrosis factor
TRAF: = TNF receptor-associated factor
TRIF: = TIR-domain-containing adapter inducing IFN-β (also called TICAM)
WT: = Wild type
